# Comparative Study of Epoxy-CsH_2_PO_4_ Composite Electrolytes and Porous Metal Based Electrocatalysts for Solid Acid Electrochemical Cells

**DOI:** 10.3390/membranes11030196

**Published:** 2021-03-11

**Authors:** Laura Navarrete, Chung-Yul Yoo, José Manuel Serra

**Affiliations:** 1 Instituto de Tecnología Química (Consejo Superior de Investigaciones Científicas–Universitat Politècnica de València), Av. Los Naranjos, s/n, 46022 Valencia, Spain; 2 Department of Chemistry, Mokpo National University, Jeollanam-do, Mokpo 58554, Korea; chungyulyoo@mokpo.ac.kr

**Keywords:** acid salts, proton conductors, fuel cells, electrolyzers

## Abstract

Electrochemical cells based on acid salts (CsH_2_PO_4_) have attracted great interest for intermediate temperature, due to the outstanding proton conductivity of acid salts. In this work, electrodes and electrolyte were optimized following different strategies. An epoxy resin was added to the CsH_2_PO_4_ material to enhance the mechanical properties of the electrolyte, achieving good conductivity, enhanced stability, and cyclability. The electrodes configuration was modified, and Ni sponge was selected as active support. The infiltration of different oxide nanoparticles was carried out to tailor the electrodes resistance by promoting the electrocatalyst activity of electrodes. The selection of a cell supported on the electrode and the addition of an epoxy resin enables the reduction of the electrolyte thickness without damaging the mechanical stability of the thinner electrolyte.

## 1. Introduction

Currently, the main energy sources are originated from fossil fuels like coal, natural gas, and oil, accounting for more than 62% of the total energy production in 2019 [1] The use of those energy sources has increased the greenhouse emissions and has induced climate change. Regarding greenhouse gases, CO_2_ is one of the pollutants more produced and has reached 68% of the total emissions generated by the industry (chemical, metallurgical, refineries, cement plants, etc.) [2]. One strategy to reduce greenhouse gases is the reduction of fossil fuels dependency. The outstanding technological development of the energy produced by renewable sources has encouraged the use of this technology as an alternative to the fossil fuels. However, the fluctuant energy production by renewable sources as wind, solar, hydro, or geothermal makes mandatory the energy storage.

H_2_ has been placed as an alternative fuel in the near future, with great possibilities due to the low CO_2_ footprint and high energy density (310 kWh/m^3^) [3]. H_2_ can be directly employed for energy production and be stored or employed as a reactant for the generation of value-added compounds. Despite the advantages from H_2_ utilization and since it is not abundant in nature, H_2_ has to be obtained by different techniques. Traditionally, it was extracted from fossil fuels through steam reforming, partial oxidation (POX), and autothermal reforming (ATR) [4]. However, large amounts of CO and CO_2_ are generated. Other processes, where the CO_2_ footprint is practically zero, are also being developed and are based on the reforming, pyrolysis, and fermentation of biomass, but the efficiencies obtained are not good enough to reduce time to market. However, H_2_ produced from water is an efficient and commercial technology with low impact on the environment. The most common techniques include thermochemical dissociation of water, photochemical processes, and electrolysis. The electrolysis of water has become a key element in future clean energy production systems, both in the generation of electricity and in the mobility sector, as well as in the chemical sector or heating processes.

Solid acid proton conductors, based on tetrahedral oxyanions, have attracted interest in the last years because of their potential applications as electrolytes in fuel cells [5], hydrogen pumping [6], or electrochemically promoted water gas shift reactions [7] or electrolyzers [8]. Solid acids of interest are those whose chemistry is based on oxyanions groups, linked together by hydrogens bonds and their charge balanced by large cations as K^+^, Cs^+^, Rb^+^, and NH_4_^+^. Cesium dihydrogen phosphate, CsH_2_PO_4_ (CDP), is one of the compounds more studied that belong to this group, since has a good compatibility with catalysts and stability [6,9,10,11]. Furthermore, H_2_-air cells based on CDP electrolytes are able to process streams containing 20% of CO [12]. CDP exhibits three different crystallographic phases depending on the surrounding temperature. At temperatures below −120 °C, it exhibits a ferroelectric phase with a monoclinic structure in space group of P2_1_. At temperatures above −120 °C, the phase changes to paraelectric with a space group P2_1_/m. CDP undergoes superprotonic conductivity due to a phase transition to cubic phase with a space group of Pm$\bar{3}$m at high temperature (above 230 ± 2 °C). The dynamically disordered hydrogen bonds and the PO_4_ disordered groups are responsible for the high proton conductivity [5] by increasing proton conductivity several orders of magnitude. Thus, CDP can be selected as good electrolyte for low temperature fuel cells or electrolyzer.

The operational temperature of the CDP allows improving some problems obtained in proton exchange membrane fuel cell (PEMFC) operation, as the water humidification control or the poisoning of the Pt for the presence of CO in the fuel stream. Furthermore, the reduction of precious metal catalyst load or even the entirely elimination of precious metals can be achieved due to the CDP operation temperature.

For instance, Haile et al. [5] obtained a power density peak of 415 mW/cm^2^ at 240 °C with hydrogen and oxygen humidified, demonstrating the applicability of CDP as electrolyte for low-medium temperatures.

In this work, CDP material has been selected as electrolyte material, for its application in fuel cells and electrolyzers. The main objective is to tailor the electrodes and electrolyte properties, conferring high-cell stability and avoiding precious metals for electrodes’ catalyst, achieving high performance. CDP was mixed with an epoxy resin to increase the mechanical stability of the electrolyte.

## 2. Materials and Methods

CsH_2_PO_4_ powder was synthesized from aqueous solutions of Cs_2_CO_3_ and H_3_PO_4_ in a molar ratio of 1:2 [8]. Afterwards, methanol was added for the precipitation of CDP. Finally, the solution was filtered and dried at 150 °C to obtain the CDP powders.

Powder after synthesis was characterized by X-ray diffraction (XRD) analysis; measurements were carried out in a Malvern Panalytical (Almelo, The Netherlands) Cubix diffractometer by using CuKα1,2 radiation, and an X’Celerator detector. XRD patterns were recorded in the 2θ range from 10 to 90° and analyzed using the X’Pert HighScore Plus software from PANalytical B.V. (Almelo, The Netherlands).

**Figure 1:**
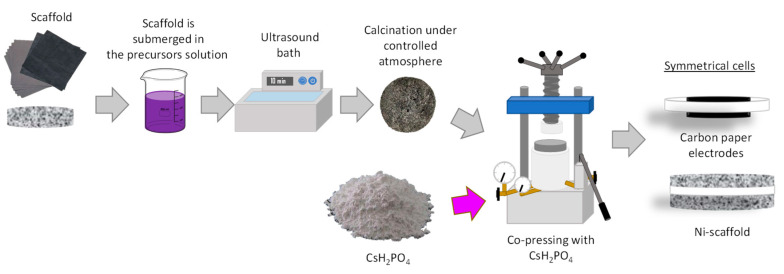
Main steps for symmetrical cells preparation.

Disk samples used for electrochemical measurements with a diameter of 13 mm were performed by uniaxial pressing. Electrodes based on Ni substrates or carbon paper were attached to both sides of the electrolyte by co-pressing the synthesized materials and two Ni substrate or carbon paper in a sandwich cell configuration, as can be observed in Figure 1.

Composite electrolytes were fabricated, by mixing with a mortar and pestle, CDP powders and the epoxy resin in different percentages of weight. The epoxy is a low-viscosity heat-resistant epoxy resin (Duralco™ 4460, Cotronics Corp. (New York, NY, USA)).

Symmetrical cells were tested by Electrochemical Impedance Spectroscopy (EIS), using a Solartron 1470E/1455 FRA (Farnborough, Hampshire, UK) with an AC potential of 20 mV and a frequency sweep from 0.03 Hz to 1MHz. The EIS measurements were performed at different operation temperatures and with different stream compositions (oxygen or hydrogen containing atmospheres). Postmortem analysis of the symmetrical cells was done by Scanning Electron Microscopy (SEM) using a JEOL JSM6300 electron microscope (Tokyo, Japan).

Electrodes were obtained by using nickel sponge as scaffolds [13]. The scaffold of carbon paper or nickel substrate was immersed in a solution with the electrocatalysts dissolved. To ensure precursors covering the whole material surface, substrates in the solution were placed in an ultrasound bath for 10 min. After removing from solution, electrodes scaffolds were dried and calcined to get the active particles. A commercial Pt/C electrode (EC1019-2, 0.5 mg/cm^2^, Naracelltech, (Seoul, Korea) was used in order to make comparison with results obtained from nickel-based electrodes.

## 3. Results

### 3.1. CDP Characterization

**Figure 2:**
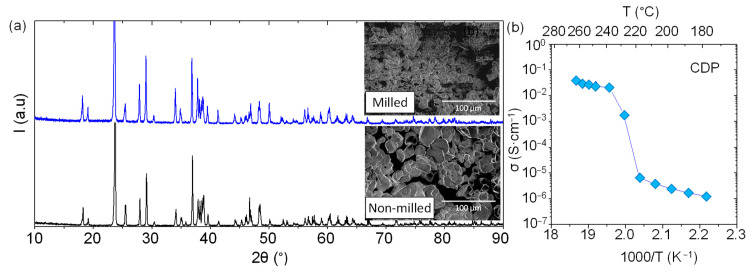
(**a**) X-Ray diffraction of cesium dihydrogen phosphate, CsH_2_PO_4_, (CDP) before and after milling 4 days and scanning electron microscopy (SEM) micrographs of CDP after synthesis and after milling. (**b**) Electrical conductivity measurements of CDP as function of temperature, with a content of 30% of water in N_2_.

The CDP material was synthesized by methanol route precipitation, after drying the powder, XRD measurements were carried out at room temperature. As can be ascribed from Figure 2 bottom, CDP material exhibits a monoclinic structure at room temperature, which was previously described by Louie et al. [14], and no extra peaks were observed in the limits detection of the XRD equipment. After CDP synthesis, the powder was milled for four days in dimethylformamide (DMF) medium. Powder was characterized before and after milling by X-Ray diffraction and SEM. The monoclinic structure of the material is maintained after milling, and not extra peaks are observed in the XRD patterns (Figure 2), and no amorphization is visible. In addition, the average particle size is reduced at least three times (Figure 2 SEM inset). 

The total conductivity of CDP salt was measured as function of temperature in a mixture of N2 and water in a 30% of content. As shown in Figure 2, there are three different regimes in the total electrical conductivity: (1) the conductivity is as low as ~2·10^−6^ S·cm^−1^, and it is related with the proton transport in the monoclinic phase; (2) there is a huge increase in terms of conductivity ascribed to the phase transition from monoclinic to cubic; and (3) the conductivity presents values higher than ~3·10^−2^ S·cm^−1^ but in the same order of magnitude. This outstanding conductivity increase of four orders of magnitude agrees with the results observed by Otomo et al. [15]. 

### 3.2. CDP and Epoxy Composites

This type of cell can work at high pressures enhancing the cell efficiency [8]; thus, the mechanical stability of the electrolyte should be enhanced. CDP exhibits high plasticity and ductility, and those properties are product of the superprotonic phase [12]. Thus, the addition of another phase can improve the mechanical stability. For instance, the addition of 10% wt. of SiO_2_ can improve the mechanical properties, i.e., reduce deformation rates [12]. Qing et al. [16] studied the influence of work with composite electrolyte, composed of CDP and an epoxy and the flexural strength, and found how is reduced by the epoxy incorporation. It has been found that 80 wt.% CDP-20% epoxy composite shows the highest proton conductivity with different CDP contents and a flexural strength of 7.3 MPa at room temperature. Even though the flexural strength of CDP-epoxy composite is similar to that of CDP -SiO_2_/C composite obtained from mixing CDP and SiO_2_/C powders, the load-crosshead displacement profile confirms that the flexibility of CDP-epoxy composite (CDP in the cross-linked epoxy matrix) is twice higher than CsH_2_PO_4_-SiO_2_/C powder composite, which can reduce the failures during the electrochemical cell fabrication.

**Figure 3:**
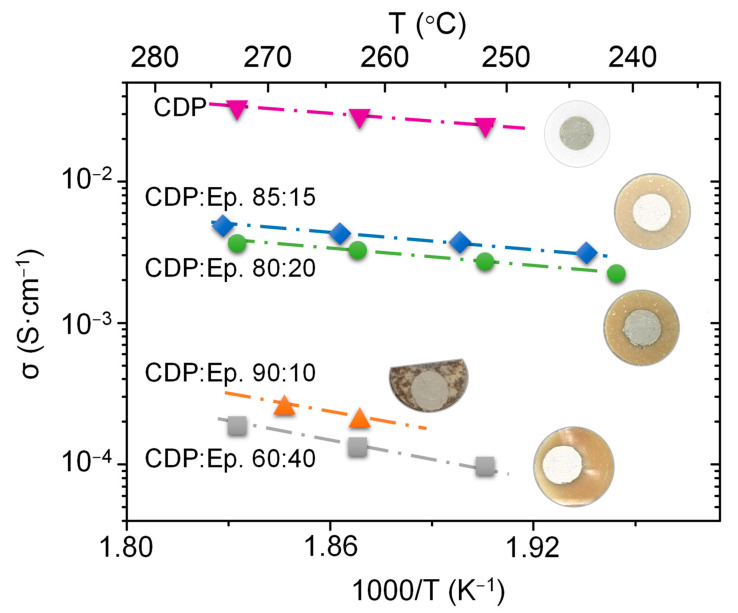
CDP: Conductivity of CDP composites extracted from electrochemical impedance spectroscopy measurements, measured in Argon with a 30% vol. of steam. Inset of samples after electrochemical test.

In this study, the composite prepared by mixing CDP and epoxy with different percentages of weight were characterized by electrochemical impedance spectroscopy in order to re-evaluate the optimized weight ratio between CDP and epoxy in the literature [17], and for that purpose, silver paste (P-100, Elcoat) electrodes were applied. Argon with a partial pressure of 0.3 bar of water was used for the EIS measurements. Results of the conductivity obtained from EIS measurements are shown in Figure 3. As can be inferred from the graph, when CDP is mixed with the epoxy the conductivity is reduced, the pure CDP exhibits the highest conductivity. The total conductivity of the electrolyte is reduced two orders of magnitude when high contents of epoxy (CDP:epoxy, 60:40) are incorporated in the electrolyte, and in the same manner, high contents of CDP have the same impact in the total conductivity. In the first case, the quantity of epoxy is too high, and proton pathways are limited due to the negligible conductivity of the epoxy (CDP:epoxy, 90:10). In the second case, the low content of liquid phase in the mixture hampers the homogenization of the sample (Figure 3). However, the samples with 80 and 85% of CDP show the best results (Figure 3), and this observation is in good agreement with the literature [16] due to the use of the identical epoxy resin.

**Figure 4:**
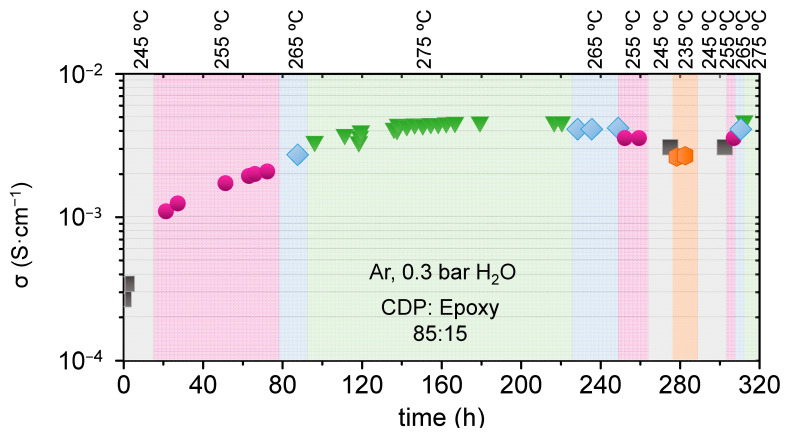
CDP: Epoxy 85:15 material conductivity measured as function of time for different temperatures with pH_2_O 0.3 bar in Ar.

The material which exhibits the highest conductivity (CDP: epoxy 85:15) was characterized during a long period of time, and results are presented in Figure 4. A stabilization period of few days is observed for samples after running the test; conductivity increases with time until a steady state is reached, and after that, temperature variation does not affect the material stability (Figure 4). As a result, for long periods of time, composite electrolytes present higher stability than bare CDP material working as electrolyte [5,17].

### 3.3. Active Electrode Support–Ni Sponge

The electrolyte conductivity has a high impact on the electrochemical performance, (i) reduces the power density of the fuel cell, and (ii) grows the operation voltage on the electrolysis cells [18]. Therefore, different strategies have been followed to reduce the electrolyte contribution. One of the most extended techniques is the deposition of the electrolyte material on a support with high mechanical stability, resulting in thin electrolytes with enhanced performance. For that purpose, in this work the design of an electrochemical cell with an active support electrode has been developed. Ni sponge electrodes designed for alkaline water electrolysis were studied as active electrodes for CDP-based cells. These types of electrodes were developed by Kim et al. [13]. Electrodes show an asymmetrical distribution in size, small pores near one surface (~5μm), and the other surface with open structure with pore size between 100–200 μm. That asymmetry allows having one layer that plays the role of electro-catalytic layer whereas the open structure allows the easy gas diffusion. Furthermore, nickel is well-known for hydrogen evolution reaction in aqueous acids and in alkaline systems [19,20] and for the same type of cells [21].

**Figure 5:**
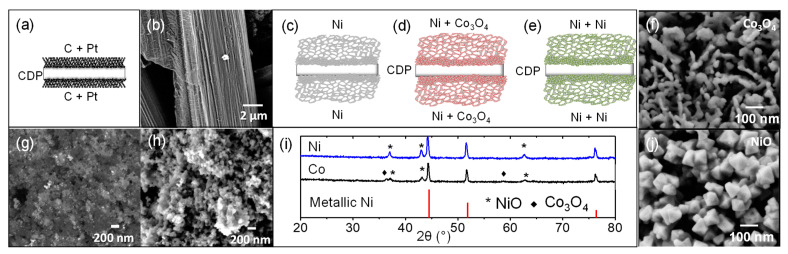
(**a**) Scheme of symmetrical cells supported on the CsH_2_PO_4_ electrolyte (CDP) configuration of commercial carbon paper infiltrated with Pt. (**b**) Field emission(FE)-SEM images of carbon fibers of carbon paper scheme of symmetrical cells of (**c**) Ni sponge, (**d**) Ni sponge infiltrated with Ni, and (**e**) Ni sponge infiltrated with CO_3_O_4_ as electrodes. (**f**) FE-SEM images of CO_3_O_4_ nanoparticles infiltrated in the Ni support. (**g**) Backscattered electrons. (**h**) Secondary electrons FE-SEM images of XC-72 with Pt nanoparticles. (**i**) XRD of nickel support infiltrated with nickel and cobalt oxides and (**j**) FE-SEM image of NiO infiltrated nanoparticles.

This type of structure provides the possibility of incorporating different catalysts with enhanced electrocatalytic properties. The catalysts can be introduced by immersion of the nickel substrate in solutions with the final catalyst precursors. Two different catalyst were studied, nickel and Co_3_O_4_. Nickel was selected since it is a good catalyst for hydrogen oxidation, and smaller particles than Ni support can have a high influence on the cell performance [22]. In order to achieve lower particle size than Ni support, lower firing temperature was selected. Co_3_O_4_ was selected since it has been employed with nickel in different types of devices with good results, as in solar technologies for hydrogen production [23] or cells for water electrolysis which operate in a KOH, showing a high performance of Ni-Co_3_O_4_ for hydrogen evolution reaction [24]. Furthermore, Ni-Co_3_O_4_ is widely used for supercapacitator applications [25]. After immersing the different nickel supports in the nickel and cobalt solutions, samples were fired at 450 °C. In order to check the presence of different nanocatalysts and phases, electrodes were characterized by XRD. As can be ascribed from Figure 5, cobalt containing sample shows three different phases, metallic nickel, NiO, and Co_3_O_4_. The metallic nickel exhibits the highest intensities whereas small peaks of NiO are observed, those peaks can be ascribed to a small oxidation of the sample in the infiltration or calcination process. Additionally, it can differentiate the peaks related with Co_3_O_4_, obtained after nitrates removal. Moreover, in the nickel oxide sample, it can distinguish only two phases, one ascribed with the nickel support and the second one that is the sum of nickel infiltrated and nickel oxide from the support.

The oxides nanoparticles obtained in the Ni support after precursor infiltration were studied by FE-SEM technique. FE-SEM images of NiO and Co_3_O_4_ nanoparticles are shown in Figure 5f–j. Co_3_O_4_ is composed of sphere-like particles and irregular particles [26] with a size below 30 nm. However, NiO presents octahedral particles [27] with a size ≈ 90 nm. The entire Ni support was covered with the different nanoparticles.

Electrodes obtained by the immersing method were characterized by electrochemical impedance spectroscopy by using symmetrical cells. Furthermore, a commercial carbon paper with a 30% of Pt loading was also measured as reference with a symmetrical cell configuration.

The carbon paper infiltrated with Pt selected for the EIS characterization was a commercial paper, 30% of Pt on XC-72 with a loading of 0.5 mg/cm^2^ supported on TGP-H-120 carbon paper from Naracelltech. FE-SEM images of the commercial electrode are presented in Figure 5. Figure 5b shows the carbon fibers of paper, with 10 μm of diameter. Figure 5g,h are two images of the same area of the electrode, but with back scattered electrons detector and with secondary electrons detector, respectively. Pt nanoparticles are well distributed, with a particle size around 2 nm. That small size of the Pt particles allows high catalytic activity [28]. In Figure 5h, the 50 nm XC-72 particles can be appreciated and present particle size below 200 nm.

**Figure 6:**
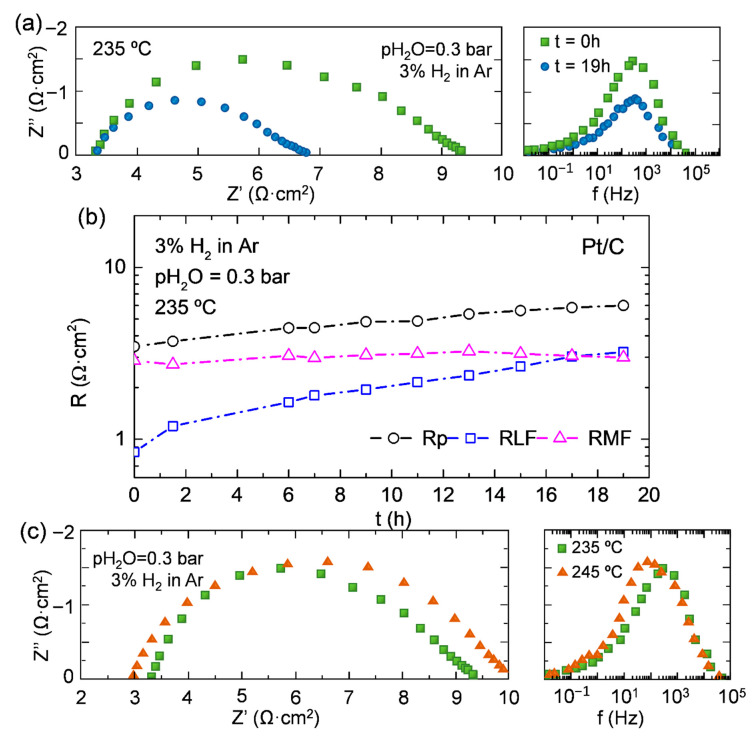
(**a**) Nyquist’s and Bode’s plots of carbon paper and (**b**) resistances obtained from equivalent electrical circuit fitting results of Pt on C paper symmetrical cell at 235 °C as function of time. (**c**) Nyquist’s and Bode’s plots of carbon paper with Pt as a function of temperature.

Results of the EIS measurements in reducing atmosphere of a symmetrical cell consisting of commercial carbon paper with Pt as electrode and CDP as electrolyte are plotted in Figure 6. As can be ascribed form the Nyquist’s and Bode’s plots, Pt exhibits an acceptable activity for the hydrogen oxidation/reduction reaction, with relatively low resistance. However, the Pt electrode is very unstable with time, after 20 h of operation polarization resistance increases to 2 Ω·cm^2^. The temperature increase has not high impact neither in the catalyst activity nor the electrode stability (Figure 6c). As can be ascribed from graphs, the biggest resistance takes place at medium frequencies (MF 10–1000 Hz) and is shifted to lower frequencies with time. To enable the possibility of checking the electrodes limitation, the EIS spectrum as function of time was fitted with an equivalent electrical circuit (LR_Ω_-(RLF-CPELF)-(RMF-CPEMF), where CPE corresponds with a Constant Phase Element, R with the different resistances associated with a characteristic process, and L is the inductance of wires and setup. Results are shown in Figure 6b. Resistance at low frequencies (RLF) increases with time whereas the MF resistance remains almost constant with time (Figure 6c). For short periods of time, the limiting resistance takes places at MF, but after 1000 min, the RLF becomes the limiting contribution, lowering the associated capacitances (Figure S1 in Supplementary Information). Ciureanu et al. [29] studied the different contributions that appeared in the EIS spectrum for Pt electrodes in PEMFC working with H_2_ atmospheres in both chambers. They found two arcs, one at high frequency and another at low frequency. The HF arc was related with the charge transfer process (Pt-H_adsorbed_ → Pt + H^+^ + e^−^) and Low Frequency (LF) with chemiadsorption of H_2_ (H_2_ + 2Pt → 2Pt-H_adsorbed_) or due to the low gas flow. Since RMF is maintained with time, it could be associated with the charge transfer, whereas for LF, the chemiadsorption would be the suitable explanation. The RLF behavior can be related with the poisoning of the Pt in contact with the CDP. The Pt poisoning reduces the activity and sites for the hydrogen chemiadsorption. A strong adsorption of phosphate anions has been reported for phosphoric acid-based cells, poisoning the platinum particles [30,31] and reducing the activity. Other degradation pathways were also observed in Pt nanoparticles by Meier et al. [32] that can be also the explanation of the electrode behavior: (i) dissolution of Pt nanoparticles in the electrolyte material, (ii) agglomeration of Pt particles, and (iii) particle detachment. Additionally, the real resistance associated with the electrolyte is kept in all measurements done at 235 °C. Even though there is degradation in the electrode performance, electrolyte decreases the resistance with temperature (Figure 6c).

**Figure 7:**
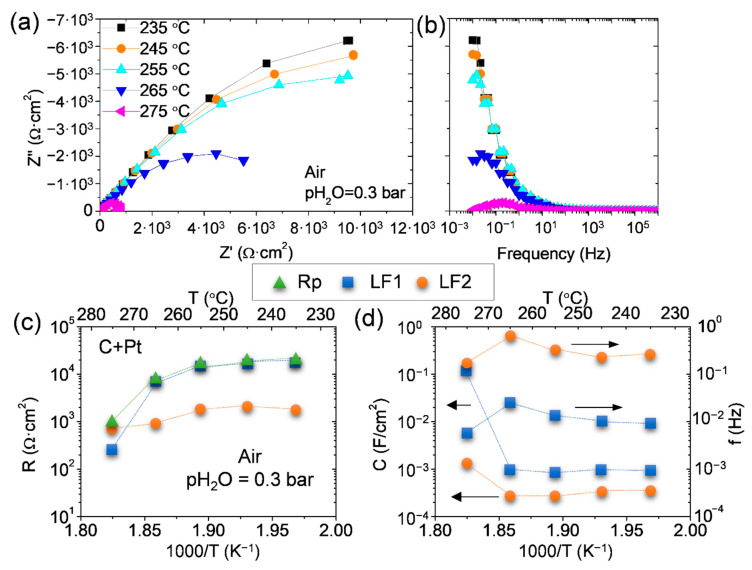
(**a**) Nyquist’s and (**b**) Bode’s plots and equivalent electrical circuit fitting results; (**c**) resistance and (**d**) capacitance and frequency of Pt on C paper symmetrical cell as function of temperature in air conditions with pH_2_O = 0.3 bar.

After EIS measurements in H_2_ atmosphere, the cell made by commercial carbon paper with Pt and CDP was tested in air atmosphere with 0.3 bar of water partial pressure. Figure 7 presents the polarization resistance at different temperatures of Pt carbon electrode. In contrast to the H_2_ results, the temperature highly affects the electrode performance with a huge activation with temperature. However, the electrode activity for the oxygen evolution reaction and oxygen reduction reaction is not as good as for the hydrogen oxidation/reduction reaction. Usually, the main limiting step in proton cells working with Pt as catalyst occurs in the cathode [33].

The oxygen reduction reaction in fuel cell mode can be described by three main stages: (1) O_2_ adsorption, (2) electrochemical and chemical reaction with intermediate species, and (3) intermediates or O_2_ protonation for the water formation [34]. The different contributions in the EIS spectra were distinguished by an equivalent electrical circuit (LR_Ω_-(RLF1-CPELF1)-(RLF2-CPELF2)). Two resistances limit the electrode performance, and both take place at low frequencies with low associated capacitances (Figure 7). RLF1 has the highest resistance contribution and reduces its resistance in more than one order of magnitude when the cell works at 275 °C. Thus, RLF1 process would be associated with the thermal activation of the Pt in the operation conditions. However, RLF2 has the lowest contribution in all temperature range, except for 275 °C, where RLF2 becomes the limiting contribution. The associated capacitances are almost constant but increases for both resistances at 275 °C. Parthasarathy et al. [35] show one big process in open circuit voltage (OCV) conditions for a PEMFC that takes place with a relaxation frequency of ≈ 0.15 Hz and could be ascribed to the oxygen-reduction charge-transfer process. This frequency range fits with the results obtained here for the RLF2. However, the RLF1 with very low associated frequencies could be attributed to the mass transport limitation [33].

**Figure 8:**
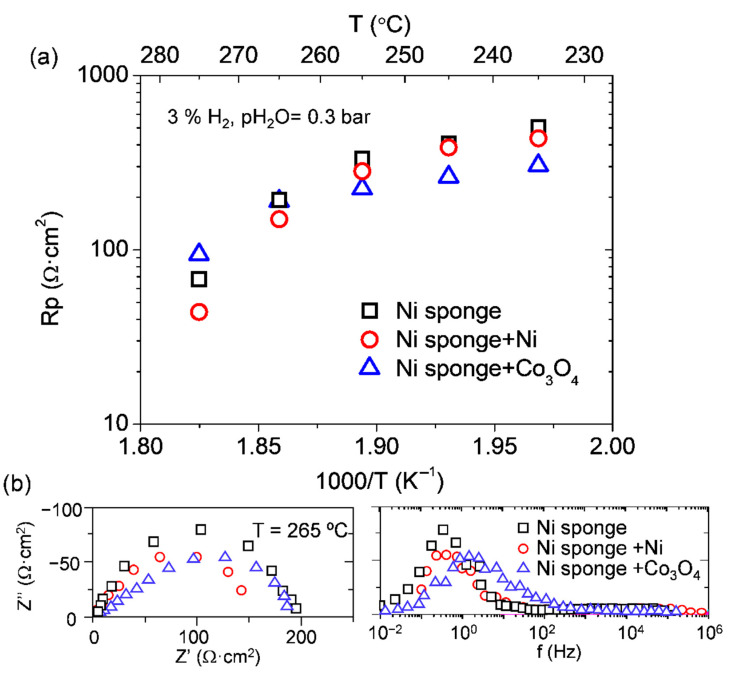
(**a**) Polarization resistance of the three different electrodes: Ni sponge, Ni sponge + Ni, and Ni sponge and Co_3_O_4_ electrodes as function of temperature. (**b**) Nyquist’s and Bode’s plot of electrodes at 265 °C.

Bare Ni sponge and infiltrated electrodes (Ni and Co_3_O_4_) were tested as electrodes in the same conditions tested for the Pt electrodes (3% H_2_ in argon with pH_2_O = 0.3 bar). The overall polarization resistance of the three different electrodes is plotted in an Arrhenius arrangement in Figure 8. The three electrodes have two different activation energies, one below 265 °C and another at higher temperatures.

All samples present a huge reduction of Rp when the temperature is increased above 265 °C. Operational temperature highly affects the electrode performance; the catalyst activation is produced, and cell improvement is deeper, at temperatures above 255 °C. Samples exhibit the same trend with temperature; due to the catalyst thermal activation, the polarization resistance is reduced as the temperature is increased. Both infiltrations can reduce the polarization resistance of the bare sample. However, the behavior of Ni and Co_3_O_4_ nanoparticles is not the same. Nickel at low temperatures has only a small contribution in the Rp improvement, whereas Co_3_O_4_ can reduce in more than 100 Ω·cm^2^ the resistance of the pristine sample. Above 275 °C, Ni infiltrated sample exhibits an outstanding improvement achieving a value of 44 Ω·cm^2^.

**Figure 9:**
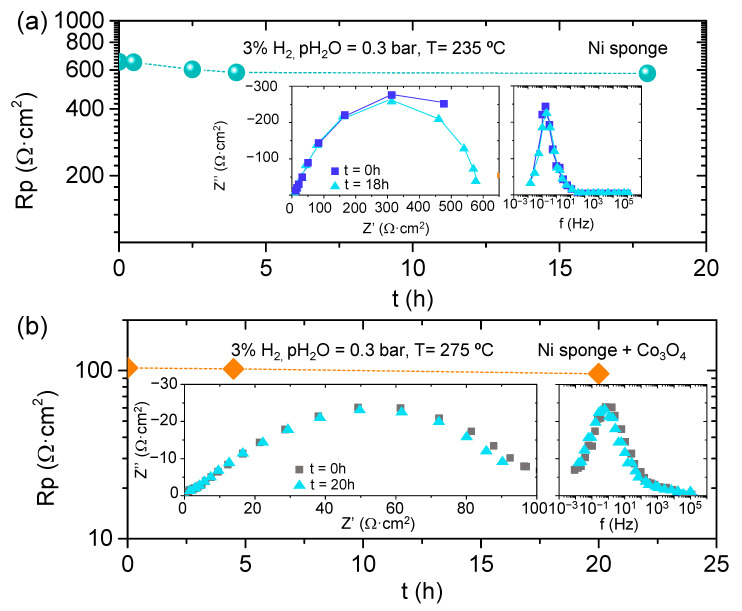
(**a**) Polarization resistance of Ni sponge/ CsH_2_PO_4_/Ni sponge cell as a function of time and Nyquist’s and Bode’s plots inset. (**b**) Polarization resistance of Ni sponge+Ni/CDP/Ni sponge+Ni cell as function of time and Nyquist’s and Bode’s plots of Ni sponge+Co_3_O_4_/CDP/Ni sponge+ Co_3_O_4_ cell.

All samples were stable along the operation time, and the Ni sponge infiltrated by Co_3_O_4_ and bare sample were checked during 20 h; the polarization resistance remains stable after more than 20 h of measurements, even reducing the polarization resistance as can be observed in Figure 9. Thus, there is no catalyst contamination or microstructure degradation. Big stability is observed for this Ni bare electrode; there is a stabilization time at the beginning of the cell operation, and 4 h of operation in steady state is achieved, with lower polarization resistance (Figure 9a). The same effect is observed for the sample infiltrated with Co_3_O_4_, and slow activation of the catalyst with time is observed in Figure 9b.

**Figure 10:**
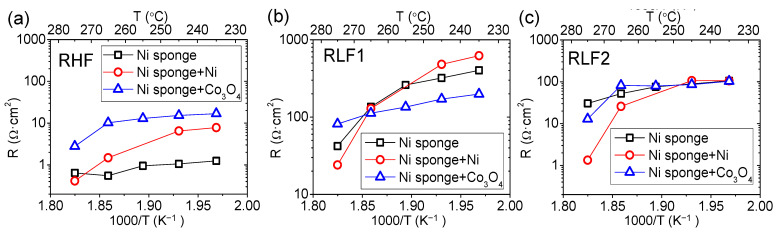
Equivalent electrical circuit fitting results: (**a**) resistances at high frequencies, (**b**) low frequency 1, and (**c**) low frequency 2 or Rchem for bare Ni sponge.

In order to go one step further, the EIS spectra at different temperatures for all Ni sponge electrodes were characterized by an equivalent circuit (Figure S2 in Supplementary Information). For the electrodes infiltrated, the equivalent electrical circuit consisted of three resistances in parallel with three CPE: LR_Ω_-(RLF1-CPELF1)-(RLF2-CPELF2)-(RHF-CPEHF). However, for the parent Ni electrode, the equivalent electrical circuit employed a Gerischer element: LR_Ω_-(RHF-CPEHF)-(RLF1-CPELF1)-G, due to the spectra shapes observed in Figure 8. This Gerischer element has to be assigned to a process coupled diffusion and surface reaction, related with the low activity of big Ni particles. To be able to compare between the different electrodes, the three processes of each electrode have been split depending on the frequency range (Figure 10). LF1 is the resistance with the lowest associated frequency; LF2 has a higher associated frequency, and HF takes place at high frequencies. In the case of the bare Ni electrode, the RLF2 refers to the R_chem_.

The lowest contribution takes place at high frequencies (2·10^3^ to 1·10^4^ Hz) with a low associated capacitance for all samples (3·10^−6^ to 2·10^−5^ F/cm^2^). The resistances for all range of temperature goes from 0.5 Ω·cm^2^ for the Ni infiltrated sample at the highest tested temperature to 20 Ω·cm^2^ for the Co_3_O_4_ at the lowest temperature. The HF resistance can be associated with the charge transfer for the hydrogen oxidation reaction (HOR) in the interface electrode-electrolyte [33,36]. Since electrodes are pure electronic conductors, the HOR will take place on the active sites located in the electrode–electrolyte surface (TPB). Co_3_O_4_ exhibits the highest resistance, whereas nickel support has the lowest ones. The higher electrical conductivity of Ni in contrast with Co_3_O_4_ could explain the lower resistance of Ni pattern electrode. In addition, there is a thermal activation in both catalysts infiltrated that can improve the charge transfer in the HOR. RLF1 process has a characteristic frequency in the range of 0.1 to 1 Hz for the three electrodes in all ranges of temperature and should be ascribed with surface processes. The associated capacitance values vary from 1·10^−3^ to 8·10^−3^ F/cm^2^. These resistances have the highest contribution in the Rp for all samples, limiting the cell performance. For all samples, there is a thermal activation in all range of temperatures. While Co_3_O_4_ shows only one activation energy in the RLF2 as function of 1000/T(Figure 10c), both Ni catalysts exhibit two activation energies. RLF2 has a frequency range between 0.5 and 20 Hz. In this case, all samples have similar resistance values until 255 °C, when there are different catalytic activations.

Different processes have been suggested for the low frequency resistances, slow gas diffusion due to the low gas flow and gas conversion or dissociative chemisorption of hydrogen at the electrode (H_2_ → 2H_adsorbed_) [29,36,37]. Since the hydrogen percentage was maintained at 3%, the lowest frequency resistance could be associated with that fact. Nevertheless, the RLF2 could be interpreted as hydrogen chemisorption and electrochemical reaction coupled, due to the frequency range and the use of Gerischer element in the Ni sponge EIS fitting. Finally, the lower resistance of Ni infiltrated catalyst at high temperatures can be assigned to the higher surface area of the smaller particles, allowing a higher active area for the hydrogen oxidation reduction reaction.

## 4. Conclusions

CsH_2_PO_4_ has been characterized by electrochemical impedance spectroscopy and XRD, showing a phase transition above 232 °C followed by outstanding conductivity improvement. As to increase the mechanical stability of the electrolyte, composite electrolytes were prepared by mixing CDP and an epoxy resin. The composite electrolyte CDP:epoxy 85:15 showed the best performance with a high stability.

In addition, Ni sponge was studied as alternative for the hydrogen oxidation reaction catalyst and support of the electrochemical cell, for fuel cell and electrolysis operation. The performance of the nickel support could be boosted infiltrating different catalysts inside the scaffold as Co_3_O_4_ and Ni. The higher surface area on the smaller Ni particles infiltrated reduces the polarization resistance of the electrode. Furthermore, the commercial carbon paper with 30% Platinum showed degradation with time, increasing the resistance of the electrode at LF. As a result, electrodes based on Ni support and tailored with different nanocatalysts can be used as alternative to the expensive and unstable Pt.

## Supplementary Materials

The following are available online at https://www.mdpi.com/2077-0375/11/3/196/s1, Figure S1: Equivalent electrical circuit fitting results: (a) resistances, (b) capacitances, and (c) frequencies for Pt carbon paper as function of time at 235 °C and 3% H_2_ and pH_2_O of 0.3 bar. Figure S2: Equivalent electrical circuit fitting results: (a) resistance, (b) capacitance, and (c) frequency at High Frequency; (d) resistance, (e) capacitance, and (f) frequency of Low Frequency 1 (LF1); (g) resistance, (h) capacitance, and (i) frequency at LF2 of Ni sponge, Ni sponge +Ni, and Ni sponge + Co_3_O_4_ at different temperatures.
