# Supplementary material for: Comparative Study of Epoxy-CsH2PO4 Composite Electrolytes and Porous Metal Based Electrocatalysts for Solid Acid Electrochemical Cells
Source: Membranes (Basel). 2021 Mar 11;11(3):196. doi: 10.3390/membranes11030196 (PMC7999483; doi:10.3390/membranes11030196)
Supplement: Supplementary file 1 [file membranes-11-00196-s001.pdf]

membranes

Supplementary Material: Comparative Study of Epoxy-CsH_2PO_4 Composite Electrolytes and Porous Metal Based Electrocatalysts for Solid Acid Electrochemical Cells

Laura Navarrete, Chung-Yul Yoo and José Manuel Serra

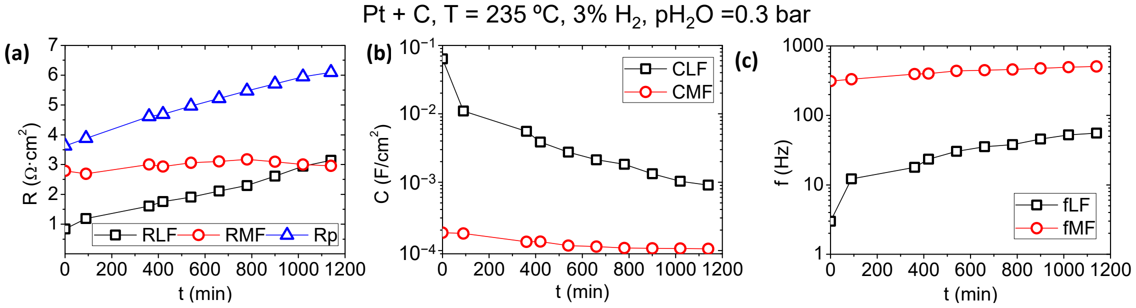

Figure S1. Equivalent electrical circuit fitting results; (**a**) resistances, (**b**) capacitances and (**c**) frequencies for Pt carbon paper as function of time at 235 °C and 3% H_2 and pH_2O of 0.3 bar.

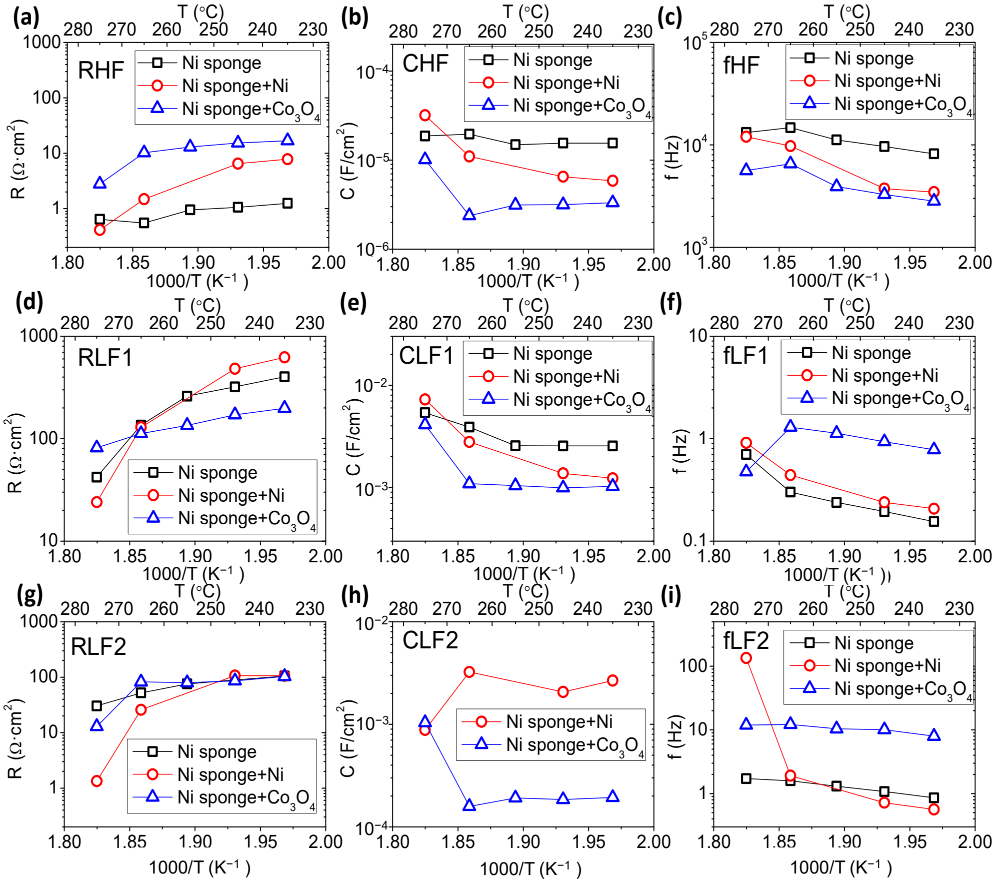

Figure S2. Equivalent electrical circuit fitting results; (**a**) resistance, (**b**) capacitance and (**c**) frequency at HF, (**d**) resistance, (**e**) capacitance and (**f**) frequency of Low Frequency 1 (LF1), (**g**) resistance, (**h**) capacitance and (**i**) frequency at LF2 of Ni sponge, Ni sponge +Ni and Ni sponge + Co_3O_4 at different temperatures.
